# Measures of falls efficacy, balance confidence, or balance recovery confidence for perturbation-based balance training

**DOI:** 10.3389/fspor.2022.1025026

**Published:** 2022-10-12

**Authors:** Shawn Leng-Hsien Soh

**Affiliations:** ^1^Health and Social Sciences Cluster, Singapore Institute of Technology, Singapore, Singapore; ^2^Centre for Health, Activity and Rehabilitation Research, Queen Margaret University, Musselburgh, United Kingdom

**Keywords:** falls efficacy, balance confidence, balance recovery confidence, self-efficacy, perturbation-based training, falls prevention, falls management, fear of falling

## Introduction

There is a growing interest in using perturbation-based balance training (PBT) to reduce falls (1). PBT is a skill training intervention that aims to improve reactive balance control in response to destabilizing perturbations in a safe and controlled environment (2). Studies have often posited that the training mechanisms of PBT improve physical abilities, such as generating more effective recovery step response and trunk movement to arrest falls in the face of a slip, trip or a loss of balance caused by volitional movement (3). This explanation has also been offered for studies employing a single PBT session (4, 5). PBT is likely to influence psychological factors. However, the impact on this aspect remains unclear. Psychological factors are well-established predictors of falls and play a role in determining performance, such as balance and gait (6). Yet, several studies have reported a limited influence of PBT on falls efficacy or balance confidence (7, 8). PBT could affect other self-efficacies, such as balance recovery confidence, safe landing confidence, or fall recovery confidence, but there are scarce studies on them. Since falls are a complex phenomenon, the concepts of the different falls-related self-efficacy (falls efficacy) constructs must be clarified. Having better clarity allows appropriate measures to be selected to elucidate the impact of PBT on the perceived ability to deal with falls.

**Figure 1:**
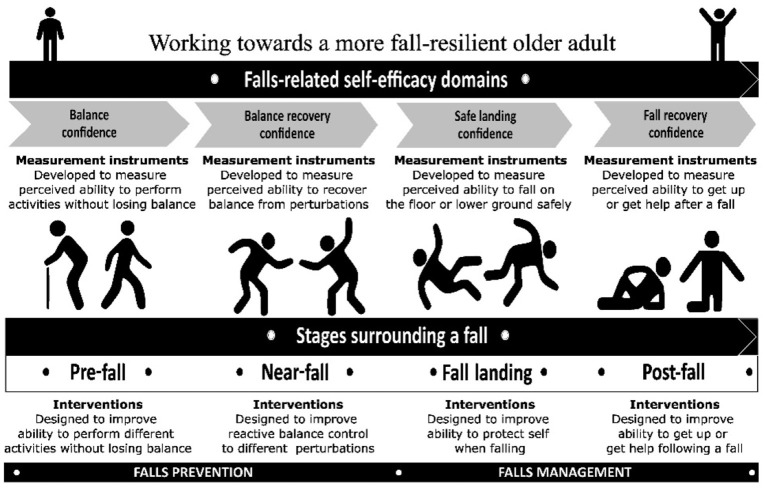
The various domains of falls efficacy and the different stages surrounding a fall. The figure has been adapted and reproduced with permission (15).

Deciphering falls efficacy has not been easy because several falls-related psychological factors have been used interchangeably in the literature. Falls efficacy is closely related to fear of falling or balance confidence, but it is necessary to recognize that these constructs are distinct (9, 10). While some research papers have presented falls efficacy and balance confidence as isomorphic (11), this paper will consider balance confidence to be a subdomain of falls efficacy. A recent methodological quality review of the content development of falls efficacy-related measurement instruments reported that falls efficacy has been viewed as a self-efficacy construct that covers different perceived abilities needed to prevent and manage falls (12). Rooted in Bandura's self-efficacy theory (13, 14), falls efficacy refers to the general belief in capabilities required to overcome various falls-related situations. This belief incorporates different self-efficacies presented across four stages surrounding falls (Figure 1) (15). In the pre-fall stage, balance confidence refers to the perceived ability to perform activities without losing balance. In the near-fall stage, balance recovery confidence focuses on the perceived ability to arrest a fall in response to destabilizing perturbations. These two stages surround the perceived capability to prevent falls (16). In the fall-landing stage, safe landing confidence relates to the perceived ability to fall safely on the ground when the balance is irrecoverable. In the post-fall stage, fall recovery confidence refers to the perceived ability to get up from the floor independently. The latter two stages surround the perceived capability to manage falls (16).

In contrast, fear of falling refers to the concerns about falling and that the individual would avoid the activity despite being able to perform (9). Fear of falling is likely to incorporate efficacy and outcome expectancies (17). Outcome expectancy is a judgement about performance outcomes, whereas efficacy expectancy is a judgement of the capability to perform in a given situation. Fear of falling measures, such as the Falls Efficacy Scale-International (18), Fear of Falling Questionnaire (19), and Fear of Falling Avoidance Behavior Questionnaire (20), do not solely assess falls efficacy expectations. Applying appropriate measurement instruments is imperative to understand PBT's role and helps reduce the risk of misinterpreting the results (21). The commentary aims to highlight some falls efficacy measures for PBT research so that researchers can make an informed decision when selecting the most suitable measures to determine perceived capabilities to deal with falls.

## Measures of falls efficacy for PBT research

Bandura's self-efficacy theory (13) states that the efficacy belief system is a differentiated set of self-efficacy beliefs linked to distinct realms of functioning. Researchers need to be clear about the intended self-efficacy beliefs or the confidence of the ability to accomplish a task or succeed in a particular situation (22). When PBT research plans for certain types of perturbations to be delivered, some balance control mechanisms and self-efficacies are predominantly targeted (23). The following examples are presented:

Example 1: Destabilizing perturbations to be delivered at an insufficient intensity to cause a fall, yet having the equilibrium perturbed adequately would likely train balance control *in situ* (23). The targeted mechanisms could be proactive, anticipatory, or reactive fixed support systems for the person to perform the task or activity more steadily, as shown in Figure 1: Pre-fall stage. Some real-world situations are standing (not holding a handrail) on a moving train or walking on a wet sidewalk. These PBT may benefit from using measures of balance confidence. The most commonly used measures are the “Falls Efficacy Scale (FES)” (24) and the “Activities-specific Balance Confidence (ABC) Scale” (25). Both scales aim to measure the confidence level to perform activities of daily living steadily. Both scales have excellent psychometric properties, such as the FES has good test-retest reliability (0.71) (24), internal consistency (0.90) (25), and scalability (0.44) (25) and the ABC scale has excellent test-retest reliability (0.92) (25), internal consistency (0.96) (25), and scalability (0.59) (25). The 10-item FES is suitable for low-functioning older adults, whereas the 16-item ABC scale is designed for higher-functioning seniors (25). Both scales are not difficult to administer, and each takes about five to ten minutes to complete.

Example 2: Large mechanical destabilizing perturbations to be delivered in such a way that insufficient or inadequate recovery reactions (i.e., reach-to-grasp or compensatory stepping) would result in a fall (1). This training aims to improve reactive change-in-support balance control, as shown in Figure 1: Near-fall stage. Real-world applications refer to individuals arresting falls in situations such as experiencing a slip when walking on a puddle of water or a trip when a foot gets caught by a curb. Such training may benefit using the measures of balance recovery confidence. One candidate measure is the Balance Recovery Confidence (BRC) Scale (26). The BRC scale measures the perceived reactive balance recovery ability in response to perturbations such as a slip, a trip or a loss of balance from volitional movement (26). The BRC scale has good psychometric properties, such as test-retest reliability (0.94) (26) and internal consistency (0.97) (26). The 19-item BRC scale has a list of pictures accompanying each item's descriptor to provide a consistent interpretation of the scenarios (26). The scale is designed for community-dwelling older adults and takes about seven to ten minutes to complete.

Example 3: PBT supplemented with other interventions, such as cognitive behavioral therapy and strength and balance exercise training, could consider multi-domain measures of falls efficacy. Multi-domain measures reveal a general sense of personal efficacy to produce certain attainment (14) and, in this context, overcome falls. This approach transcends the separate subdomains, as noted in Figure 1, where a more meta-efficacy measure could demonstrate an overall change in falls efficacy. One candidate measure is the Perceived Ability to Prevent and Manage Falls Risks (PAPMFR) scale (27). The six-item PAPMFR scale aims to measure confidence in the ability to prevent and manage falls. Items included: “Steadiness on their feet”, “Balance while walking”, “Ability to walk in their homes”, “Ability to walk outdoors”, “Ability to prevent falls”, and “Ability to find a way to get up if they fall”. The PAPMFR scale was conceptually designed to measure the perceived ability to deal with falls. The scale has good psychometric properties, such as excellent internal consistency (0.94), good structural validity and construct validity (27). The PAPMFR scale is developed for community-dwelling older adults and takes about 5–7 min to complete.

There are lacking measures for other constructs, such as safe-landing confidence (Figure 1: Fall landing stage) and fall recovery confidence (Figure 1: Post-fall stage). Selecting items from multi-domain measures may be considered but should be done circumspectly. One item is the “Protect yourself if you fall” from the Perceived Ability to Manage Risk of Falls or Actual Falls scale (28) for safe-landing confidence (Figure 1: Fall-landing stage). Another is the “Ability to find a way to get up if they fall” from the PAPMFR scale (27) for fall recovery confidence (Figure 1: Post-fall stage). However, these measures have not been rigorously validated, unlike the FES or the ABC scale. Researchers must be cautious when using these measures or selecting certain items to evaluate specific constructs or falls efficacy. There is an urgent need for validation studies to critically evaluate these measures using the COSMIN methodology (29) to present their psychometric properties (i.e., content development and validity, structural validity, construct validity, reliability, responsiveness, measurement error).

## Discussion

Bandura's self-efficacy theory has been an enduring concept for understanding behavior outcomes and would be applicable for PBT in falls prevention and management. The self-efficacy theory explains how efficacy expectations can determine whether coping behaviors will be initiated, how much effort will be expended, and how long the self-efficacy will be sustained in the face of obstacles and adverse experiences (30). PBT research must clarify the self-judged efficacy of interest when designing different perturbation strategies to help older people overcome falls. In other words, which of the constructs, such as the overall confidence to prevent and manage falls (falls efficacy), or the specific constructs, such as the balance confidence, balance recovery confidence, safe landing confidence, and fall recovery confidence, are being targeted? The most suitable measure should then be applied. Potentially, PBT could address the fear of falling by having graded perturbations prescribed with the starting perturbations set at lower strengths of self-judged efficacy. Appropriate identification of the targeted self-efficacy allows PBT to be planned appropriately for individuals to achieve performance mastery and build their self-efficacy (31). Previous studies have shown that falls efficacy plays a mediator between fear and functional abilities (32, 33). PBT could be purposefully designed to alleviate fear by enhancing falls efficacy and achieving improved performance such as balance and gait.

Given that there are varying capabilities to deal with falls, researchers need to discern the objectives of the PBT. Measures of falls efficacy could be employed in various ways. Some researchers may be keen to use PBT to address falls efficacy and thus apply the measures as outcome tools to evaluate the effectiveness of the intervention. Others may wish to use PBT to address the fear of falling and activity-related avoidance behaviors using the self-efficacy theory. Falls efficacy measures can then act as a conduit to inform the design of the PBT's perturbations. For example, the BRC scale contains 19 different “potential near-fall” scenarios depicting a range of perturbations-types (e.g., a slip or a trip), direction-specific (e.g., forward or backward), environmental constraints (e.g., indoor or outdoor), and set-ups for balance recovery strategies (e.g., availability of handrail or uneven ground level). The BRC scale can help researchers plan suitable perturbations by identifying challenging scenarios reported by certain groups of individuals.

Falls efficacy measures should be used alongside other assessments in PBT research to understand perceived and actual abilities. Unlike observable parameters such as kinematic changes, reactive skill performances or reduction in falls, latent psychological factors require researchers to be explicit about the construct of interest. Selecting the most appropriate measures is imperative to elucidate the psychological impact of PBT to help older people overcome falls (9). Moreover, a greater use of appropriate fall efficacy measures in PBT research allows “patient-centered” data captured to demonstrate measurable and meaningful improvements (34). Presenting the perceived capabilities of the individual in real-world falls-related scenarios will provide empirical evidence that the effects of PBT are translatable from a simulated environment to real-life generalization.

## Author contributions

The author confirms being the sole contributor of this work and has approved it for publication.

## Conflict of interest

The author declares that the research was conducted in the absence of any commercial or financial relationships that could be construed as a potential conflict of interest.

## Publisher's note

All claims expressed in this article are solely those of the authors and do not necessarily represent those of their affiliated organizations, or those of the publisher, the editors and the reviewers. Any product that may be evaluated in this article, or claim that may be made by its manufacturer, is not guaranteed or endorsed by the publisher.

## Author disclaimer

The opinions given in this article are those of the author and do not necessarily represent the official position of the universities listed.
